# Fear of COVID-19: Data of a large longitudinal survey conducted between March 2020 and June 2021

**DOI:** 10.1016/j.dib.2023.109177

**Published:** 2023-04-25

**Authors:** Gaëtan Mertens, Paul Lodder, Tom Smeets, Stefanie Duijndam

**Affiliations:** a Department of Medical and Clinical Psychology, Tilburg University, PO box 90153, 5000 LE Tilburg, the Netherlands; b Department of Methodology and Statistics, Tilburg University, PO Box 90153, 5000 LE Tilburg, the Netherlands

**Keywords:** Fear, COVID-19, Longitudinal, Vaccination, Anxiety, Personality traits, Media, Coping

## Abstract

Research indicates that fear was an important factor in determining individual responses to COVID-19, predicting relevant behaviors such as compliance to preventive measures (e.g., hand washing) and stress reactions (e.g., poor sleep quality). Given this central role of fear, it is important to understand more about its temporal changes during the COVID-19 pandemic. This article describes a publicly available dataset that contains longitudinal assessment of fear of COVID-19 and other relevant constructs during the first 15 months of the pandemic. Particularly, the dataset contains data from two different samples. The first sample consists predominantly of Dutch respondents (*N* = 439) who completed a cross-sectional survey in March 2020. The second sample consists of a large-scale longitudinal survey (*N* = 2000 at T1), including respondents with a broad range of nationalities (though predominantly residing in Europe and North America; 95.6%). The respondents of the second sample completed the survey between April 2020 and August 2020 using the Prolific data collection platform. In addition, one follow-up assessment was completed in June 2021. The measures included in the survey were fear of COVID-19, demographic information (age, gender, country of residence, education level, and working in healthcare), anxious traits (i.e., intolerance of uncertainty, health anxiety, and worrying), media use, self-rated health, perceived ability to prevent infection, and perceived risk for loved ones. Additionally, at the follow-up assessment in June 2021, respondents were asked whether they were vaccinated against COVID-19 or were planning to get vaccinated. The datafiles of this study have been made available through the Open Science Framework and can be freely reused by psychologists, social scientists, and other researchers who wish to investigate the development, correlates, and consequences of fear of COVID-19.

**Specifications Table**

**Table:** 

Subject	Psychology
Specific subject area	Cross-sectional and longitudinal survey research on Fear of COVID-19
Type of data	Survey response data, including: - Raw datafiles (Excel data files format) - Processed datafiles (SPSS data files format)
How the data were acquired	Data were acquired through a (repeated) web-based survey in two waves of data collection. During the first wave, respondents were recruited via social media platforms (e.g., LinkedIn, Facebook, Twitter and Reddit) and during the second wave respondents were recruited through the Prolific platform (https://www.prolific.co/). The survey was delivered online, through the Qualtrics platform (https://qualtrics.co/). An overview of the included questionnaires is included in the Supplementary Materials. Due to potential copyright infringements, the complete list of the items of the questionnaires is not provided.
Data format	Raw Microsoft Excel datafiles. Analyzed SPSS datafiles
Description of data collection	Data were acquired through an online survey between March 2020 and August 2020, and one follow-up datapoint in June 2021. Each month, between the 13^th^/14^th^ and 17^th^ day of the month, respondents were re-invited to complete the survey. Respondents received a link to the survey through social media platforms (March) or through the prolific platform (April 2020-June 2021). The survey was presented to respondents in English. The only inclusion criterion was that participants are sufficiently fluent in English. There were no exclusion criteria.
Data source location	Institution: Tilburg University City/Town/Region: Tilburg Country: the Netherlands
Data accessibility	Repository name: Open Science Framework Data identification number (doi):10.17605/OSF.IO/RYNDG Direct URL to data: https://osf.io/ryndg/ Instructions for accessing these data: The data are freely available under a Creative Commons by Attribution (CC-By Attribution 4.0 International) license. They can be directly downloaded from the above link.
Related research article	Mertens, G., Lodder, P., Smeets, T., & Duijndam, S. (2023). Pandemic panic? Results of a 14-month longitudinal study on fear of COVID-19. *Journal of Affective Disorders*, 322, 15-23. https://doi.org/10.1016/j.jad.2022.11.008

## Value of the Data

• This sample contains longitudinal data of 7 key-moments during the COVID-19 pandemic. The data can be used to identify predictors of fear of COVID-19 and fear of disease in general.
• The data can be used to get an insight in the course of fear of COVID-19, and several other psychologically relevant constructs such as intolerance of uncertainty, worrying, health anxiety, perceived health, perceived risk control, perceived risk for loved ones and voluntary media exposure during the COVID-19 pandemic.
• The respondents in the current samples reside in different regions of the world. These data can be used to compare anxious reactions during the COVID-19 pandemic in residents in different regions of the world.
• Data in the described samples can be used to identify constructs and mechanisms that are important for clinicians and policy makers to manage psychological aspects of future pandemics.
• The data can be used in educational settings, for instance by demonstrating data analysis techniques (e.g., structural equation modelling, longitudinal data analysis) or for student projects (e.g., data for a master's thesis).

## 1 Data Description

The data were collected with the aim of investigating the development of fear of COVID-19 during the pandemic. The data presented in this article consist of two samples and were collected across seven time points, between March 2020 and June 2021. The first sample consisted of 439 respondents (predominantly Dutch, 47.6%), who completed the survey between the 13^th^ and 17^th^ of March 2020. The second sample consisted of an international group of respondents (n = 2000) recruited through the prolific platform (https://www.prolific.co/), who completed six waves of data collection between April 2020 and June 2021. The participants were re-invited between the 14^th^ and 17^th^ day of each month to complete the survey again. Thereafter, respondents were re-invited once again between the 14^th^ and 17th of June 2021 as a follow-up and to additionally get information about their COVID-19 vaccination status. The survey was always presented in English. From the second sample, 1050 participants (52.5%) completed all measurements between April 2020 and August 2020 and 668 participants (33.4%) completed all measurements, including the last one in June 2021. The sample size of the two samples was based on practical considerations (i.e., available budget) and maintaining a large enough sample size (>250) for stable correlations after attrition [1]. A more detailed overview of the demographics in the samples is provided in Table 1.

**Table 1:** Overview of the demographic information for the two samples of respondents included in the available datafiles.

	First sample: March 2020 (*N*=439)		Second sample: April 2020 to June 2021 (*N*=2000)^1^	
	*N*	*%*	*N*	*%*
**Age in years**				
11-20	46	10.5%	315	15.8%
21-30	215	48.0%	887	44.4%
31-40	98	22.3%	478	23.9%
41-500	47	10.7%	184	9.2%
51-60	16	3.6%	90	4.5%
61-70	16	3.6%	42	2.1%
71-80	1	0.2%	4	0.2%
**Gender**				
Male	126	28.7%	1018	50.9%
Female	307	69.9%	972	48.6%
Prefer not to say	6	1.4%	10	0.5%
**Highest education**				
Less than High School	22	5.0%	47	2.4%
High School diploma	34	7.7%	796	39.8%
College degree	63	14.4%	802	40.1%
Master's degree	277	63.1%	320	16.0%
Doctorate (PhD or equivalent)	43	9.8%	35	1.8%
**Region of residence**^2^				
Asia	3	0.7%	5	0.3%
Oceania	4	0.9%	37	1.9%
Europe	321	73.1%	1419	71.0%
Middle-East	2	0.5%	11	0.5%
North-America	102	23.2%	497	24.9%
South-America	7	1.6%	15	0.8%
Sub-Sahara Africa	0	0%	16	0.8%
**Work in healthcare**				
Yes	48	10.9%	95	4.8%
No	345	78.6%	1859	93.0%
Unsure	46	10.5%	46	2.3%
**Infected COVID-19**				
Yes	0	0%	17	0.9%
No	392	89.8%	1871	93.6%
Unsure	47	10.7%	112	5.6%

1 Information in this table is based on the data from April 2020.

2 Full list countries of residence: Australia, Austria, Belgium, Canada, Chile, Cyprus, Czech Republic, Denmark, Estonia, Finland, France, Germany, Greece, Hong Kong (S.A.R.), Hungary, India, Ireland, Israel, Italy, Japan, Latvia, Mexico, Netherlands, New Zealand, Norway, Peru, Poland, Portugal, Romania, Russian Federation, Slovenia, South Africa, South Korea, Spain, Sweden, Switzerland, Turkey, United Arab Emirates, United Kingdom, USA.

## 2 Experimental Design, Materials and Methods

**Fig. 1:**
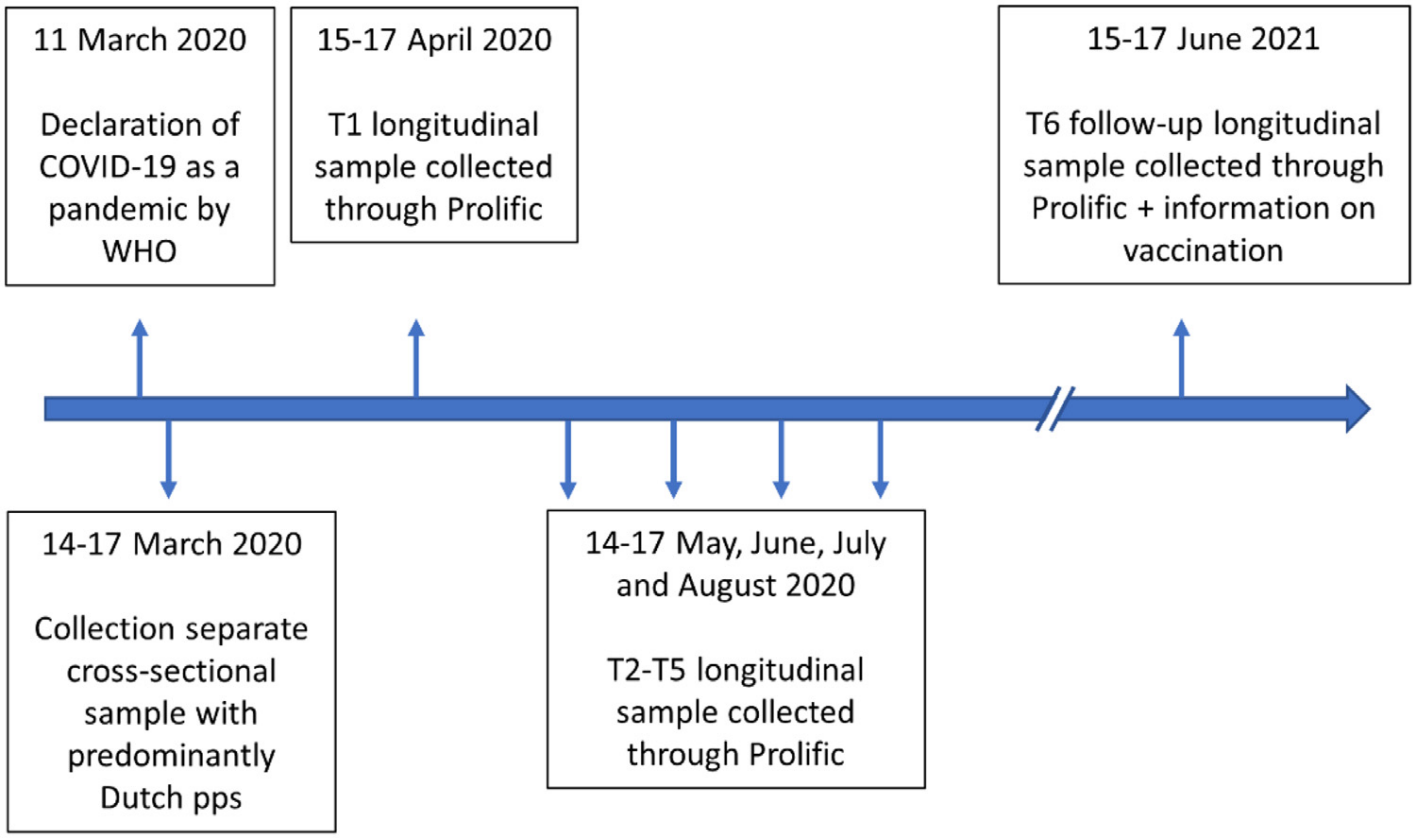
Overview of the timeline of the data collection and the different samples included in the dataset. At each measurement timepoint, participants were asked to report on the fear of COVID-19, intolerance of uncertainty, the tendency to worry, health anxiety, exposure to COVID-19 related news, general health, risk control, risk for loved ones, demographic information, and vaccination status (the latter was only assessed at the last data point). Note that two separate samples were included in the study and dataset: one cross-sectional sample of mostly Dutch participants recruited right after the declaration of the COVID-19 outbreak as a pandemic by the WHO. The second sample consisted of participants from the Prolific work platform that were re-invited to participate in a longitudinal panel design between April 2020 and June 2021. Abbreviations: pps = participants; WHO = World Health Organization.

The design of the study was a longitudinal panel design with seven time points (see Fig. 1), of which the first time point had a separate sample which was matched to the other sample based on the demographic characteristics gender, age, education level, and region of origin.

Samples presented here were recruited via multiple means. For the first cross-sectional sample, respondents were recruited through online advertisements using social media platforms and via the networks of the involved researchers (e.g., LinkedIn, Facebook, Twitter, Reddit). For the second longitudinal sample, respondents were recruited through the Prolific platform (https://www.prolific.co/). The Prolific platform is an online working platform of more than 100 thousand active participants. Typically, it only takes a few hours to collect several thousands of participants to answer a questionnaire. The initial 2000 respondents of the longitudinal study were re-invited each month through the Prolific website to continue to participate in our study. Please note that this sampling methodology relied on convenience sampling and that the participants in our study are therefore not necessarily representative of the general population of the countries we collected data from.

The only inclusion criterion for both samples was that participants are sufficiently fluent in English. This was implied in the recruitment text for the first sample (i.e., it was mentioned that the survey was in English) and selected as a prescreening criterion for the second sample in Prolific. There were no exclusion criteria.

In both samples, questionnaires were delivered through an online survey using the Qualtrics platform (https://www.qualtrics.com/). The survey could be completed in approximately 15 minutes, by using a personal computer/laptop, tablet, or smartphone. Respondents were not compensated during the first wave of data collection, during the second wave respondents were compensated according to the standard rate of Prolific at the time of data collection (£7.5/h).

The survey contained measures to assess fear of COVID-19, intolerance of uncertainty, the tendency to worry, health anxiety, voluntary exposure to COVID-19 related news, general health, risk control, risk for loved ones, demographic information, and vaccination status (the latter only being assessed at the last data point). The included variables are briefly described below. A complete list of all the included items in the survey can be found in the Supplementary Materials.

### 2.1 Fear of COVID-19

Fear of COVID-19 was assessed using the Fear of the Coronavirus Questionnaire (FCQ) developed by Mertens et al. (2020). This is an 8-item questionnaire measuring different dimensions of fear, in which respondents were asked to rate their level of agreement with each statement on a 5-point Likert scale, ranging from 1 = “Strongly disagree” to 5 = “Strongly agree”. Examples of statements are: “I am very worried about the corona virus outbreak.” and “I am worried that friends or family will be infected.”. A total score can be calculated by summing the items, resulting in a possible scoring range of 8 to 40, with higher scores indicating more Fear of the Coronavirus. Previous research indicated this to be a reliable scale (Cronbach's alpha = 0.75-0.80) [[2], [3], [4]].

### 2.2 Open ended question about main concerns related to COVID-19

To supplement the assessment of Fear of COVID-19 with the FCQ (Mertens et al., 2020), we also included an open-ended question to qualitative information about respondents; main concerns related to COVID-19. Particularly, respondents were asked the following question: “Please describe briefly your biggest concern about coronavirus”, which they could answer using an open-ended response format.

### 2.3 Intolerance of uncertainty

The shortened version of the Intolerance of Uncertainty Scale (IUS-12) was used to measure intolerance of uncertainty [5]. This questionnaire assesses an individual's tendency to find uncertain situations unpleasant. Respondents were asked to answer 12 statements on a 5-point Likert scale, ranging from 1 = “Not at all characteristic of me” to 5 = “Entirely characteristic of me”). Examples of statements are: “Uncertainty keeps me from living a full life.” and “I can't stand being taken by surprise.” The total score can be calculated by summing the item scores. Higher scores indicated greater intolerance of uncertainty. The IUS-12 was developed and validated by Carleton et al. (2007), they found it to be a sound measure, representing anxious and avoidance components of intolerance of uncertainty.

### 2.4 Tendency to worry

The shortened version of the Penn State Worry Questionnaire (PSWQ) was used to measure an individual's general tendency to worry [6]. This questionnaire consisted of eight items that were scored on a 5-point Likert scale, ranging from 1 = “Not at all typical of me” to 5 = “Very typical of me”. Examples of items are: “Many situations make me worry.” and “I am always worrying about something.”. A total score can be calculated by summing the items, with higher scores indicating a greater propensity to worry.

### 2.5 Health anxiety

The Short Health Anxiety Inventory (SHAI) was included in the survey to measure individuals’ tendency to worry about their health [7]. This questionnaire consists of 18 questions with four-choice answer options. Examples include: 1 = “As a rule I am not afraid that I have a serious illness.” to 4 = “I am always afraid that I have a serious illness.” and 1 = “If I notice an unexplained bodily sensation I don't find it difficult to think about other things.” to 4 = “If I notice an unexplained bodily sensation I always find it difficult to think about other things.”. A total score can be calculated by summing the item scores, with higher scores indicating a higher tendency to worry about health. Abramowitz et al. (2007) found the SHAI to be a reliable and valid measure for assessing cognitive domains of health anxiety in healthy individuals.

### 2.6 General health, risk control and risk for loved ones

Respondents were asked to answer a question about their general health: “Overall, I would rate my general health as:”. Answering options range from “Extremely good” to “Extremely bad”, on a 5-point Likert scale. Perceived risk control was assessed by the following question: “Overall, I believe that I can control or avoid becoming infected by the coronavirus (e.g., by limiting social contact, washing hands, wearing a face mask, etc.). This question could be answered on a 5-point Likert scale, with options ranging from “Strongly agree’ to “Strongly disagree”. Finally, risk perception for loved ones was assessed by asking to answer the question: “Overall, I believe that people that I care about (e.g., grandparents) are at risk of becoming infected and seriously ill due to the coronavirus outbreak. Answering options were provided on a 5-point Likert scale, ranging from “Strongly agree’, “Somewhat agree” to “Strongly disagree”. Note that the items were not based on previously published literature but were constructed for the specific purpose of this study. Nonetheless, it is useful to note that single-item measures are becoming more prevalent in psychological science and can be valid measures for specific unidimensional constructs [8,9].

### 2.7 Media exposure

To assess voluntary exposure to news about COVID-19, respondents were asked to answer the following question: “Have you looked up any extra information regarding the coronavirus outbreak? (not taking into account coincidentally seeing/reading about it in the news)”. This question could be answered with “yes” or “no”. If respondents had looked up any information, they were asked to indicate what sources they consulted. Examples of options were: “Regular newspapers/websites/TV news”, “Social media (Facebook, Twitter, Instagram, etc.)”, and “Professional websites (health institute, blogs posted by virologists/biologists, etc.)”. Multiple answers were possible. Thereafter, respondents were asked if they paid attention to the source of the media outlet when looking up new information on a 5-point Likert scale, ranging from 1 = “Strongly agree”, 5 = “Strongly disagree”.

### 2.8 Vaccination status (June 2021 data point only)

Respondents were asked to provide information about their vaccination status by answering the following question: “Have you been vaccinated against COVID-19?”. The response options were: “Yes (first vaccine)”, “Yes (fully vaccinated)”, “No (I did not receive an invitation to get vaccinated yet)”, “No (I do not want to get vaccinated)”, “Other (please explain)”.

### 2.9 Demographic information

Lastly, demographic information was asked from the respondents. They were asked about the gender they identify with the most, whether they suffer from chronic illnesses, their age (in decade categories), their highest education level obtained, whether they work in healthcare, whether they already got infected by the virus and their country of residence.

## Ethics Statements

Ethical approval was obtained from the Ethics Committee of the Tilburg School of Social and Behavioral Sciences (reference code: RP216). Respondents’ participation was on a voluntary basis and all participants provided informed consent.

## CRediT authorship contribution statement

**Gaëtan Mertens:** Conceptualization, Data curation, Funding acquisition, Investigation, Methodology, Project administration, Supervision, Writing – original draft. **Paul Lodder:** Data curation, Supervision, Writing – review & editing. **Tom Smeets:** Resources, Supervision, Writing – review & editing. **Stefanie Duijndam:** Data curation, Project administration, Writing – review & editing.

## Declaration of Competing Interest

The authors declare that they have no known competing financial interests or personal relationships that could have appeared to influence the work reported in this paper.

## Data Availability

Tracking fear levels for the coronavirus (COVID-19) (Original data) (Open Science Framework).

## Appendix Supplementary materials

Image, application 1

## References

[1] Schönbrodt F.D., Perugini M. (2013). At what sample size do correlations stabilize?. J. Res. Pers..

[2] Mertens G., Gerritsen L., Duijndam S., Salemink E., Engelhard I.M. (2020). Fear of the coronavirus (COVID-19): predictors in an online study conducted in March 2020. J. Anxiety Disord..

[3] Mertens G., Duijndam S., Smeets T., Lodder P. (2021). The latent and item structure of COVID-19 fear: a comparison of four COVID-19 fear questionnaires using SEM and network analyses. J. Anxiety Disord..

[4] Vos L.M.W., Habibović M., Nyklíček I., Smeets T., Mertens G. (2021). Optimism, mindfulness, and resilience as potential protective factors for the mental health consequences of fear of the coronavirus. Psychiatry Res..

[5] Carleton R.N., Norton M.A.P.J., Asmundson G.J.G. (2007). Fearing the unknown: a short version of the Intolerance of Uncertainty Scale. J. Anxiety Disord..

[6] Meyer T.J., Miller M.L., Metzger R.L., Borkovec T.D. (1990). Development and validation of the penn state worry questionnaire. Behav. Res. Ther..

[7] Abramowitz J.S., Deacon B.J., Valentiner D.P. (2007). The short health anxiety inventory: psychometric properties and construct validity in a non-clinical sample. Cognit. Ther. Res..

[8] Allen M.S., Iliescu D., Greiff S. (2022). Single item measures in psychological science. Eur. J. Psychol. Assess..

[9] Bergkvist L., Rossiter J.R. (2007). The predictive validity of multiple-item versus single-item measures of the same constructs. J. Mark. Res..

