# Supplementary material for: Fear of COVID-19: Data of a large longitudinal survey conducted between March 2020 and June 2021
Source: Data Brief. 2023 Apr 25;48:109177. doi: 10.1016/j.dib.2023.109177 (PMC10127663; doi:10.1016/j.dib.2023.109177)
Supplement: Supplementary file 1 [file mmc1.docx]

Supplementary Materials for:

“Fear of COVID-19: Data of a large longitudinal survey conducted between March 2020 and June 2021”

Authors:

Gaëtan Mertens^1^

Paul Lodder^1, 2^

Tom Smeets^1^

Stefanie Duijndam^1^

Affiliations:

^1^Department of Medical and Clinical Psychology, Tilburg University

^2^Department of Methodology and Statistics, Tilburg University

Corresponding author’s contact details:

^1^Department of Medical and Clinical Psychology, Tilburg University

PO Box 90153, 5000 LE Tilburg, the Netherlands

Email: [g.mertens@tilburguniversity.edu](mailto:g.mertens@tilburguniversity.edu)

**Supplementary Materials 1: Overview of the included items and questionnaires in the survey**

Fear of the Coronavirus Questionnaire (8 items) (Mertens et al., 2020)

Open-ended question biggest concern about the coronavirus:

- “Please describe briefly your biggest concern about coronavirus”

Question about mental healthcare related to the coronavirus:

- “Are you currently seeing or planning to see a psychologist or psychiatrist for any mental health issues relating to the coronavirus?”
  - Yes (currently seeing a psychologist/psychiatrist)
  - Yes (planning to see a psychologist/psychiatrist)
  - No

Intolerance of uncertainty scale – short version (12 items) (Carleton et al., 2007)

Penn State Worry Questionnaire (16 items) (Meyer et al., 1990)

Health anxiety inventory (short version; 18 items) (Abramowitz et al., 2007)

Chronic illness question

- “Do you suffer from any chronic illness?”
  - Yes (please specify)
  - No

Media exposure questions (3 questions)

- “Have you looked up any extra information regarding the corona virus outbreak? (not taking into account coincidentally seeing/reading about it in the news)”
  - Yes/No
- “What is your main source for information regarding the corona virus outbreak?”
  - No source/not looking up information
  - Regular newspapers/websites/TV news, Social media (Facebook, Twitter, Instagram, …)
  - Professional websites (health institute, professional blogs by virologists/biologists, …)
  - Friends/family/acquaintances
  - Other (please specify)
  - Did not look up any information
- “If you indicated 'yes' on the previous questions, did you pay any attention to the source of the information? If you answered 'no' on the previous question, you can select 'does not apply'.”
  - 1 = strongly agree, 7 = strongly disagree / 8 = does not apply

Age (0-100)

Gender

- “What is the gender you identify with most?”
  - Male
  - Female
  - Prefer not to say

Self-reported health

- “Overall, I would rate my general health as”:
  - 7-point Likert: 1 = extremely good, 7 = extremely bad

Control of infection

- “Overall, I believe that I can control or avoid becoming infected by the coronavirus (e.g., by limiting social contact, washing hands, wearing a face mask, etc.):”
  - 5-point Likert: 1 = strongly agree; 5 = strongly disagree

Risk for loved ones

- “Overall, I believe that people that I care about (e.g., grandparents) are at risk of becoming infected and seriously ill due to the coronavirus outbreak:”
  - 5-point Likert: 1 = strongly agree; 5 = strongly disagree

Education level

- “What is the highest level of school you have completed or the highest degree you have received?”
  - Less than high school degree
  - High school graduate (high school diploma or equivalent including GED)
  - Some college but no degree
  - Associate degree in college (2-year)
  - Bachelor's degree in college (4-year)
  - Master's degree
  - Doctoral degree
  - Professional degree (e.g., Medical Doctor)

Working in health care

- “Do you work in health care (e.g., as a doctor, nurse) or a closely related field?”
  - Yes (as a doctor)
  - Yes (as a nurse)
  - Yes (as support/technical staff)
  - No
  - Unsure (please clarify)

Already infected

- “Did you already get infected by the coronavirus (i.e., either currently being treated/recovering, or already recovered)?”
  - Yes
  - No
  - Unsure (please clarify)

Vaccination status

- “Have you been vaccinated against COVID-19?”
  - Yes (first vaccine)
  - Yes (fully vaccinated)
  - No (I did not receive an invitation to get vaccinated yet)
  - No (I do not want to get vaccinated)
  - Other (please explain)

Country of residence

Extra suggestions

- “Do you have any comments, thoughts or suggestions regarding this study?”
  - Open ended answering option

**References**

Abramowitz, J. S., Deacon, B. J., & Valentiner, D. P. (2007). The Short Health Anxiety Inventory: Psychometric Properties and Construct Validity in a Non-clinical Sample. *Cognitive Therapy and Research*, *31*(6), 871–883. https://doi.org/10.1007/s10608-006-9058-1

Carleton, R. N., Norton, M. A. P. J., & Asmundson, G. J. G. (2007). Fearing the unknown: A short version of the Intolerance of Uncertainty Scale. *Journal of Anxiety Disorders*, *21*(1), 105–117. https://doi.org/10.1016/j.janxdis.2006.03.014

Mertens, G., Gerritsen, L., Duijndam, S., Salemink, E., & Engelhard, I. M. (2020). Fear of the coronavirus (COVID-19): Predictors in an online study conducted in March 2020. *Journal of Anxiety Disorders*, 102258. https://doi.org/10.1016/j.janxdis.2020.102258

Meyer, T. J., Miller, M. L., Metzger, R. L., & Borkovec, T. D. (1990). Development and validation of the penn state worry questionnaire. *Behaviour Research and Therapy*, *28*(6), 487–495. https://doi.org/10.1016/0005-7967(90)90135-6
